# Validation of a cost-effective alternative for a radiochromatography method to be used in a developing country

**DOI:** 10.1186/s41181-020-0092-1

**Published:** 2020-04-10

**Authors:** F. P. Ekoume, H. H. Boersma, F. Dong à Zok, S. M. Rubow

**Affiliations:** 1 Department of Nuclear Medicine, Yaoundé General Hospital, P.O. Box 5408, Yaoundé, Cameroon; 2 Nuclear Medicine Division, Stellenbosch University, P.O. Box 241, 8000 Cape Town, South Africa; 3 Department of Nuclear Medicine and Molecular Imaging, University Medical Center Groningen, Hanzeplein 1, P.O. Box 30.001, 9700RB Groningen, The Netherlands

**Keywords:** Radiochemical method, Quality control, Developing countries

## Abstract

**Introduction:**

The radiochemical purity (RCP) of technetium-99m labelled radiopharmaceuticals (RP) is important to ensure optimal scintigraphic image quality. In low-income settings, it may not be possible to use compendial analytical methods or expensive equipment for radiochemical purity analysis. All radiochemical analysis methods should however be validated against compendial or otherwise proven methods. To ensure the efficacy of RP prepared at Yaoundé General Hospital (YGH) Cameroon, this study cross-validated a cost-effective routine chromatographic method using a simple survey meter technique. A GMP-compliant method used at the University Medical Center Groningen (UMCG), the Netherlands was used as the comparator.

**Methods:**

Sestamibi, HMDP and DMSA kits currently used at YGH were reconstituted at UMCG with about 2000 MBq of freshly eluted sodium pertechnetate as described by the manufacturer, and spiked with eluate of the same generator to obtain a range of impurity concentrations. Samples of technetium-99m RP were spotted on 1 × 10 cm iTLC-SG strips and developed in appropriate mobile phases. Each strip was first scanned on the chromatogram-scanner used at the UMCG (standard method), and immediately thereafter the strip was cut in two pieces and radioactivity from each portion was counted with a small survey meter from YGH. The percentage RCP for each TLC strip was calculated using both counting methods. Internationally recommended validation parameters and acceptance criteria were used. Student’s paired t-test or ANOVA were used with ‘no significant difference’ designated at a 95% confidence-interval (*P* ≥ 0.05). Linearity of the survey meter was determined for Tc-99m. Readings obtained with the survey meter were also plotted against the scanner results.

**Results and discussion:**

The proposed method proved to be accurate (CV of mean RCP < 2), precise (RSD < 2%), linear (slope close to 1, *r*^2^ ≥ 0.99) within the RCP range of approximately 80% to 100%, and robust (*P* > 0.05). LOD and LOQ were determined for the survey meter. Specificity depends on chemical separation. As we were validating the suitability of a method to quantify radioactivity, specificity was not included in the validation parameters.

**Conclusion:**

The proposed method compared well with the standard method and is suitable as a reliable low cost method for limited resource settings.

## Introduction

### Importance of method validation

Nuclear Medicine needs optimal quality radiopharmaceuticals for accurate diagnosis and therapy of diseases (IAEA 2008; Dondi et al. 2011). As the use of Nuclear Medicine increases in developing countries, including those in sub-Saharan Africa, so does the need for radiopharmaceuticals. With the increasing demand for radiopharmaceuticals, it is of paramount importance to ensure that only safe and effective products are administered to patients (IAEA 2016). Even though most radiopharmaceuticals in sub-Saharan Africa are prepared in small scale units, quality assurance programs should be implemented (Elsinga et al. 2010). Internationally, guidelines for Good Radiopharmacy Practice recommend that only validated analytical methods should be used to evaluate product quality before patient use. Quality assurance (QA) in the production of radiopharmaceuticals ensures that they are of sufficient quality for their intended purpose; it reduces the possibility of producing substandard products by adhering to specified procedures and protocols (Vincenti et al. 2016). Analytical Quality Control (QC) methods form part of quality assurance, helping to assess the quality of the product. According to Ajay et al., validation of analytical methods provides evidence that the method is suitable for it intended use, and thus helps to ensure provision of safe and effective final products (Ajay and Rohit 2012). The consequence of using unvalidated non-compendial methods could result in the administration of unknown substances, poor quality images and/or an unnecessary radiation exposure of patients (Amin et al. 2011; Vincenti et al. 2016).

### The principle of validation of quantitative analytical methods

Validation of an analytical procedure is the process to ensure that the performance characteristics of the procedure adopted in a particular laboratory meet the requirements for the intended analytical application (USP 37 < 1225> 2008). In radiopharmacy, one of the most frequently used analytical criteria is radiochemical purity (RCP) of the product. The radiochemical purity of a preparation is the fraction of the total radioactivity in the desired chemical form of the radiopharmaceutical. During the labelling process, radiochemical impurities may be present as a result of incomplete radiolabelling or decomposition products due to the presence of oxidizing or reducing agents, radiolysis or change of temperature and pH (Saha 2010; Millar et al. 2009; Loveless 2009; Mambilima 2016).

RCP can be assessed by various analytical techniques, for example paper, thin layer, or liquid chromatography and electrophoresis. Radiochromatography involves the separation of components in solution (depending on their affinity with the chromatography materials), and the measurement of the distribution of radioactivity on the chromatogram (Saha 2010).

International guidelines describe validation parameters and acceptance criteria for validation of analytical methods, including accuracy, precision, intermediate precision, linearity, range, specificity, robustness, detection limit and quantitation limit (USP 37 < 1225> 2008; EDQM 2018; Krause 2003).

### Validation studies of radiochemical analysis methods in literature

In the literature, analytical methods’ performance characteristics for both SPECT (Single Photon Emission Computed Tomography) and PET (Positron Emission Tomography) radiopharmaceuticals differ slightly from one study to another. The parameters recommended by the ICH (International Council for Harmonisation 2006), namely accuracy, precision, linearity and range, specificity, robustness and limit of quantitation (LOQ), are frequently used, especially for HPLC (High-Performance Liquid Chromatography) methods. However, it should be noted that radio-TLC and radio-HPLC express RCP as a ratio between counts or areas, without requiring absolute quantitative measurement of either the product or impurities. In this scenario, accuracy and LOQ, which both address quantitative measurement, may not be relevant for radio-HPLC or radio-TLC (Todde et al. 2014), or may need a different approach than in the case of chemical analyses.

Mihon et al. mention the ICH parameters for the HPLC determination of identity and RCP of [^18^F]NaF (Mihon et al. 2016). Leonardi et al. use these parameters in the validation of a paper chromatography method as an alternative for determination of the RCP of [^18^F]NaF (Leonardi et al. 2012). Seetharaman et al. (2006) validate their simplified solid phase extraction method for determining the RCP of [^99m^Tc]Tc-MAG_3_ by determining specificity, linearity, and robustness. They compare their method against a standard method to evaluate accuracy and precision. Acceptance criteria for analytical method validation may require adjustments according to the type of equipment: radio-HPLC, radio-UPLC (Ultra Performance Liquid Chromatography), radio-TLC (Thin Layer Chromatography), or gamma spectrometry (Todde et al. 2017). Besides performance characteristics related to the method of analysis, analytical method validation could differ regarding experimental details such as mobile phase, stationary phase, flow rate, and wavelength of UV detectors (Todde et al. 2017).

Several studies describe the development of alternative chromatography procedures to replace ‘gold-standard’ methods for reasons including simplified technical handling and more rapid processing times. Faria et al. in 2015 studied an alternative chromatographic system for the quality control of MIBI where they evaluated different solvents to optimize separation efficiency, reproducibility and analysis time (Faria et al. 2015). This was, however, not a full validation study as defined in documents of normative character: ISO (International Organization for Standardization), ICH, FDA (Food and Drug Administration) (Krause 2002; USP 37 < 1225> 2008), with performance characteristics evaluated against acceptance criteria from references. Luebke et al. (2000) also evaluated an alternative testing method for the radiochemical purity determination of MIBI but did not include all parameters recommended in guidelines (USP 37 < 1225> 2008). Something similar was done for ^99m^Tc-Annexin A5, using a binding assay instead of radio-TLC for determination of radiochemical purity as a release method (Boersma et al. 2004).

A variety of methods for quantification of distribution of radioactivity on radio-TLC plates are described in literature. Decristoforo and Zolle provide an overview of seven methods used for ^99m^Tc radiopharmaceuticals, including cutting and counting in a scintillation counter, chromatogram scanning, analysis by linear analyzer and phosphor-imager autoradiography (Decristoforo and Zolle 2007). The methods differ regarding sensitivity, resolution and linearity, but differences in time required per analysis and the cost of equipment of each of these methods are considerable, making their adoption in resource-poor developing countries challenging. At Yaoundé General Hospital in Cameroon (YGH) there is no chromatogram scanner or more advanced equipment with which the distribution of radioactivity or chromatograms of radiopharmaceuticals can be analyzed. An alternative method was therefore sought, namely counting sections of the chromatography strips corresponding to the distribution of the different radiochemical species with a contamination monitor available in the Nuclear Medicine department at YGH. Prior to employing it in daily practice, this proposed method had to be validated. The work described herein was performed to validate a cost-effective method for quantifying the distribution of radioactivity on chromatography strips using a RadEye 20 contamination monitor (available at YGH), and comparing it to the already validated method routinely used at the UMCG in the Netherlands.

## Materials and methods

### Materials for product preparation and chromatography

All experiments were performed at the UMCG radiopharmacy in the Netherlands. SPECT radiopharmaceutical kits Stamicis (2-methoxyisobutylisonitrile: MIBI), Osteocis (hydroxymethylene diphosphonate: HMDP), and Renocis (dimercaptosuccinic acid: DMSA), all from Curium, France, currently used for nuclear medicine imaging at YGH, were used for this study. Technetium-99m (^99m^Tc) was obtained from an Ultra-Technekow generator (Mallinckrodt Medical BV, Netherlands). TLC scanning was performed using a VCS-103 scanner (Comecer S.P.A., Italy) used at UMCG. A calibrated RadEye B20 multipurpose counter (Thermo Fisher Scientific Messtechnik, Germany) was brought from YGH and used at UMCG for the comparative experiments. Chromatography was done using iTLC-SG sheets (Varian) with butan-2-one (methyl ethyl ketone) (Fluka) or 0.9% saline solution (B Braun) as mobile phases.

### Sample preparation

Freshly eluted technetium-99m sodium pertechnetate ([^99m^Tc]NaTcO_4_) was used to prepare each radiopharmaceutical following the kit manufacturer’s instructions or summary of product characteristics (Osteocis 3 mg 2016; Renocis 2016; Stamicis 2016). Technetium-99 m-radiopharmaceutical kits ([^99m^Tc]Tc-HMDP, [^99m^Tc]Tc-MIBI and [^99m^Tc]Tc-DMSA) each from a single lot were prepared and spiked with adequate quantities of sodium pertechnetate in order to reach different RCP values to evaluate. The radiochemical purity we aimed for in the spiked products ranged between approximately 80% and 100%.

Each of the cold kits included in this study was reconstituted with about 2000 MBq [^99m^Tc]NaTcO_4_^−^ read with the dose calibrator (VDC 404 Veenstra, Joure).

### Chromatography of radiopharmaceuticals

A drop of the same Tc-99 m eluate used for kit reconstitution (and to spike the radiopharmaceuticals) was used to run a control analysis under the same conditions as those of the radiopharmaceutical samples.

5 μl of [^99m^Tc]Tc-RP samples was spotted on 10 cm iTLC-SG strips. For HMDP and DMSA, the strips were then developed in methyl ethyl ketone as mobile phase, and for MIBI, the strips were developed in NaCl 0.9%. Under these conditions, [^99m^Tc]Tc-HMDP, [^99m^Tc]Tc-DMSA and [^99m^Tc]Tc-MIBI remained at the origin and the impurity (pertechnetate) migrated with the solvent front. After developing, each strip was first scanned on the Veenstra VCS-203 TLC scanner using a low energy collimator with energy window range from 135 to 145 keV. The scan time was 60 s and background counts were subtracted. Within 5 min thereafter the strip was cut in 2 pieces at 4 cm from the origin. The radioactivity from each portion of the strip was counted for 1 min with the RadEye B 20 counter which is a multipurpose survey meter with gamma energy range from 17 keV to 3 MeV (measurement range from 0 to 500 kcps). Each strip was placed flat in the bottom of a 10 cm deep container and the counter placed at the top of the container to ensure counting geometry was the same for all samples. Each count rate reading was recorded with background count rate subtracted. From these values, the percentage of radiochemical purity of each strip was calculated.

### Validation studies

The methods for validation of determination of RCP of the current study followed international guidelines from the United States Pharmacopoeia (USP 37 < 1225> 2008), and the EDQM Guide (2018). Parameters used include accuracy, precision repeatability, intermediate precision, linearity, assay range, method robustness, limit of detection (LOD) and limit of quantitation (LOQ). Note that specificity of chromatographic methods depends on the chemical separation of compound and impurities. In the validation of the radioactive counting method, this parameter is thus not relevant.

#### Accuracy

Mean RCP values from three replicates of each sample were determined as well as the coefficient of variation (CV). The accuracy criteria for the radiopharmaceutical component are met when the mean of the standard method differed by not more than 1.5% from the mean value of the proposed method.

#### Repeatability

The repeatability precision was assessed with five replicates of a spiked sample and the relative standard deviation (RSD) was calculated.

#### Intermediate precision

The intermediate precision was determined by repeating at two different time points (3 h between the tests), the chromatography of 5 replicates of spiked samples performed by 2 different operators, quantifying distribution of activity on the strips using the two different counting systems. Mean RCP values and RSD were calculated. The statistical evaluation by ANOVA of the complete data set where results are grouped by each operator, each time point and instrument were analyzed with acceptance criteria stating no significant difference at 95% CI (*P* ≥ 0.05).

#### Linearity

To test the linearity, the response of the survey meter to Tc-99 m was evaluated. A 5 MBq point source of Tc-99 m was repeatedly counted over a period of 27 h. The measured count rate was plotted against the calculated activity of the point source. In addition, chromatography of three replicates from each spiked sample were performed and the chromatograms were read with both the scanner and the counter for RCP determination as described above. The linearity results include the equation from the linear regression obtained from a plot of % RCP determined with the counter (proposed method) against the RCP values determined with the scanner (the validated control method of the GMP compliant radiopharmacy), its slope, the correlation coefficient and Y intercept. As acceptance criteria, the slope should be close to 1, and *r*^2^ ≥ 0.99.

#### Robustness

**Fig. 1:**
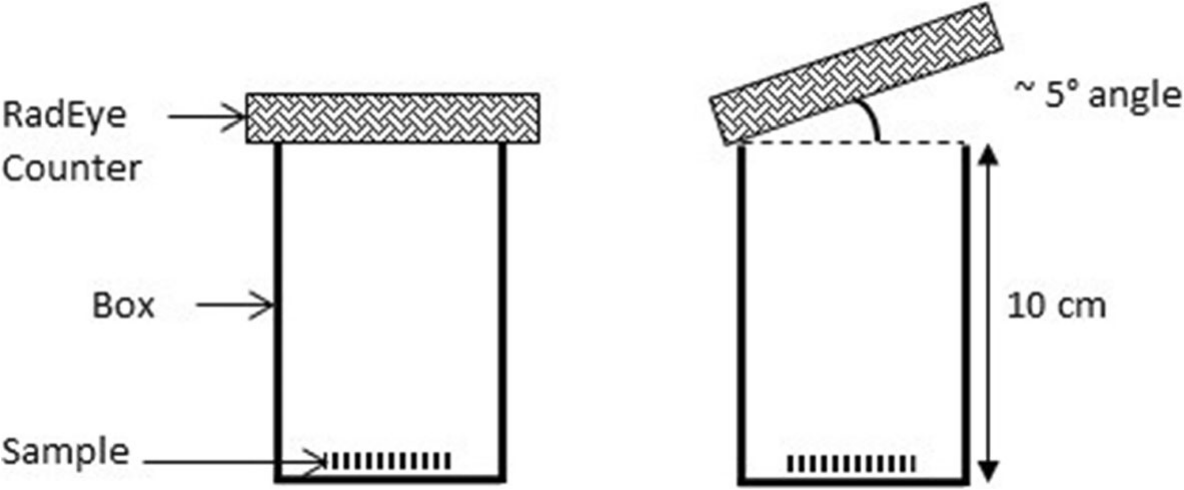
Illustration of the robustness test methodology (reading with counter at normal position and at 5° angle)

The robustness of the chromatography counting method with the RadEye B20 counter was evaluated by comparing the variation of count rates of five replicates of a spiked sample of MIBI. The pairs of strip sections were read with the counter in slightly different positions, i.e. one in the normal reading position and the other section with the counter at an angle differing 5° from the normal position (Fig. 1). Mean RCP from each set of counts and RSD were calculated. ANOVA was used to check if there were any significant differences between the groups. Acceptance criteria are RSD < 5% and *P* ≥ 0.05.

#### Limit of detection and limit and quantitation

The limit of detection of the RadEye B20 counter was evaluated by counting a blank (strip with no analyte) twenty times with the counter and calculating the mean of blank counts plus 3 times the standard deviation of the blank. The limit of quantitation was calculated as the mean of blank counts plus 10 times the standard deviation of the blank (EDQM 2018).

## Results

Complete results are shown in tables and figures.

### Accuracy

Samples with RCP between 78% and 100%, with activities between 2 and 3 MBq were spotted on the TLC plates. The mean RCP of triplicate analyses at each sample RCP level from reading of both instruments for the 3 radiopharmaceuticals are shown in Table 1.

**Table 1:** Accuracy of the proposed method for the 3 compounds

DMSA %RCP Mean ± CV			MIBI %RCP Mean ± CV			HMDP %RCP Mean ± CV		
Scanner	Counter	Diff	Scanner	Counter	Diff	Scanner	Counter	Diff
99.7 ± 0.1	99.5 ± 0.1	0.2	99.3 ± 0.4	99.2 ± 0.1	0.1	99.9 ± 0.0	99.4 ± 0.2	0.5
99.2 ± 1.1	99.2 ± 0.1	0.0	98.5 ± 0.4	98.8 ± 0.7	0.3	97.8 ± 1.0	98.0 ± 1.0	0.2
92.0 ± 1.7	91.8 ± 1.1	0.2	91.3 ± 3.7	90.6 ± 3.0	0.7	96.6 ± 1.0	95.2 ± 0.9	1.4
87.9 ± 1.6	88.1 ± 1.3	0.2	84.7 ± 0.6	86.0 ± 2.0	1.3	87.1 ± 1.2	86.3 ± 1.1	0.8
82.9 ± 0.8	81.9 ± 1.5	1.0	78.9 ± 5.0	78.4 ± 7.0	0.5	82.0 ± 0.6	82.1 ± 0.8	0.1

*N* = 3; Diff: difference between RCP from scanner and counter for each product; *CV* coefficient of variation.

### Repeatability precision

The RSD for 5 replicates of spiked samples for the three products from both devices varied between 0.6% and 1.7%. For the impurity values, the RSD ranged from 0.5 to 6.1 (Table 2).

**Table 2:** Repeatability precision of the proposed method for the 3 compounds

	DMSA % RCP Mean ± RSD		MIBI % RCP Mean ± RSD		HMDP % RCP Mean ± RSD	
	Scanner	Counter	Scanner	Counter	Scanner	Counter
RCP	83.1 ± 0.6	83.4 ± 0.8	79.7 ± 1.7	80.9 ± 1.4	82. 0 ± 0.7	82.0 ± 0.6
Impurity	16.8 ± 3.6	16.6 ± 4.2	19.1 ± 4.3	19.0 ± 6.1	18. 0 ± 3.1	17.9 ± 0.5

*n* = 5; *RSD* relative standard deviation.

### Intermediate precision

Results from the method performed by different operators at different time points for the three products gave RCP range from 78.3% to 83.4%, impurity range from 16.8% to 23.3%, highest RSD value 6.1 with P values between 0.2 and 11.8 (ANOVA) (Tables 3 and 4).

**Table 3:** Intermediate Precision results: Different Operators (*n* = 5, acceptance criteria *P* ≥ 0.05)

Spiked samples	Device	Operator 1 Mean %RCP ± RSD	Operator 2 Mean %RCP ± RSD	ANOVA (P)
DMSA	Scanner RCP	83.1 ± 0.6	81.6 ± 0.8	3.1
	Scanner Imp	16.8 ± 3.6	18.1 ± 2.9	0.7
	Counter RCP	83.4 ± 0.8	82.5 ± 0.7	0.5
	Counter Imp	16.6 ± 4.2	17.4 ± 3.7	0.5
MIBI	Scanner RCP	79.7 ± 1.7	79.8 ± 1.0	1.1
	Scanner Imp	19.1 ± 4.3	19.6 ± 4.9	0.6
	Counter RCP	80.9 ± 1.4	80.3 ± 0.7	0.8
	Counter Imp	19.0 ± 6.1	19.6 ± 2.9	0.8
HMDP	Scanner RCP	82.0 ± 0.7	81.6 ± 0.9	0.4
	Scanner Imp	18.0 ± 3.1	18.0 ± 4.7	0.4
	Counter RCP	82.4 ± 0.6	82.3 ± 0.5	0.2
	Counter Imp	17.9 ± 0.5	17.6 ± 3.1	0.2

**Table 4:** Intermediate Precision results: Effect of Time (acceptance criteria ANOVA P ≥ 0.05)

Spiked samples	Device	Time 1 mean %RCP ± RSD	Time 2 mean %RCP ± RSD	P
DMSA	Scanner RCP	83.1 ± 0.6	78.3 ± 0.8	5.8
	Scanner Imp	18.8 ± 3.6	20.1 ± 4.5	3.2
	Counter RCP	83.4 ± 0.8	76.7 ± 1.4	11.8
	Counter Imp	16.6 ± 4.2	23.3 ± 4.9	11.8
MIBI	Scanner RCP	79.7 ± 1.7	77.2 ± 1.1	2.6
	Scanner Imp	19.1 ± 4.3	22.6 ± 4.5	3.4
	Counter RCP	80.9 ± 1.4	78.2 ± 0.6	2.5
	Counter Imp	19.0 ± 6.1	21.7 ± 2.3	2.5
HMDP	Scanner RCP	82.0 ± 0.7	79.1 ± 1.1	2.5
	Scanner Imp	18.0 ± 3.1	19.9 ± 4.4	1.3
	Counter RCP	82.0 ± 0.6	78.3 ± 0.9	3.8
	Counter Imp	17.9 ± 0.5	21.6 ± 3.4	3.8

Note: Same operator at different times; *n* = 5; Imp: impurity

### Linearity

**Fig. 2:**
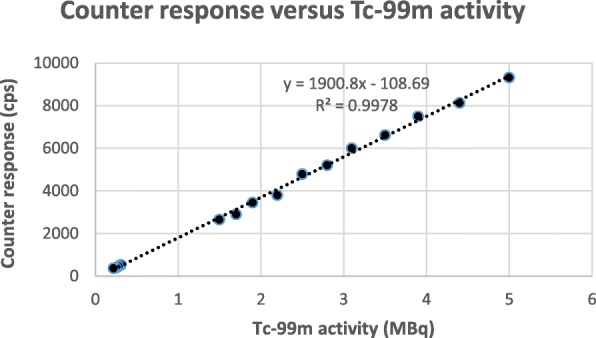
Response of the counter for Tc-99m activity

**Fig. 3:**
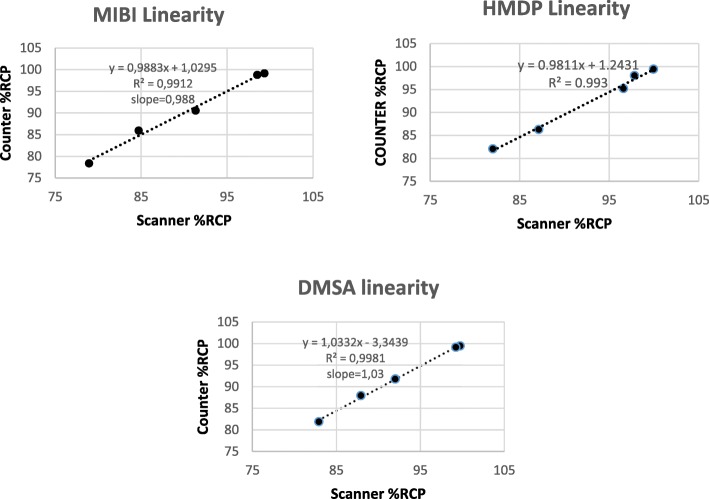
Linearity of the proposed method for the 3 products, counter response compared with RCP values determined with the scanner

The response of the counter to Tc-99m activity ranging from 0.2 MBq to 5 MBq is shown in Fig. 2. The regression curves for values obtained with the survey meter against the scanner results for each of the radiopharmaceuticals are shown in Fig. 3. All values of *r*^2^ were above 0.99 and all slopes close to 1.

### Robustness

Results from 5 replicates of a spiked MIBI sample with one part counted with slight deviation in counter position compared very well with those obtained with the counter in the normal position Results are given in Table 5.

**Table 5:** Robustness of the counter

	%RCP Mean ± RSD	%RCP Mean ± RSD	%RCP Mean ± RSD
Change	counter at normal position for both parts	counter tilted 5° for the strip section containing the impurity	counter tilted 5° for the strip section with the bound compound
MIBI (*n* = 5)	91.5 ± 1.0	91.8 ± 2.6 (*P* = 2.9*)	91.9 ± 2.3 (*P* = 2.4*)

*P values for ANOVA of the respective RCP compared to RCP obtained with the counter in the normal position

### Limit of detection and limit of quantitation

The results from twenty counts of the blank sample (*n* = 20) gave 0.6 counts per second as a mean value of the blank with standard deviation of 0.1, for a LOD value of 0.9 counts per second. The calculated LOQ was 1.6 counts per second. Based on the linearity curve this represents approximately 0.06 MBq.

## Discussion

### The importance of method validation

Recommended analytical methods are supplied with package inserts from radiopharmaceutical kits and by pharmacopoeia monographs. In radiopharmacies with limited funding, it may be difficult to follow the recommended analytical methods due to lack of adequate equipment and limited availability of consumables. Other disadvantages of many of the recommended methods include the time required to complete the tests, which delays administration of the short-lived products to patients, and use of relatively large volumes of solvents when solid phase extraction (SPE) or HPLC are used (Seetharaman et al. 2004). Thus there is a clear need for practical, simple, faster (Hammes et al. 2004; Mihon et al. 2016) and low cost methods. Any alternative methods should be validated prior to use (Leonardi et al. 2012).

The current study describes the validation of a cost-effective quantification method for instant thin layer radiochromatography strips using a counting system (RadEye 20) available at YGH, cross-validated with a routine method used at the GMP compliant radiopharmacy at UMCG.

In the current work, the validation parameters prescribed by the ICH (USP 37< 1225> 2008), i.e. linearity, precision, intermediate precision, accuracy, assay range, method robustness, limit of detection and limit of quantitation were all included for each of 3 SPECT radiopharmaceuticals commonly used at YGH (HMDP, DMSA, MIBI).

### Standards and sampling

In validation of analytical methods, reference standards are generally used for comparison purpose. Certified reference standards for ^99m^Tc impurities do not exist in the radiopharmaceutical industry due to the short half-life and short shelf life of the radiolabeled complexes as well as absence of a stable isotope of ^99m^Tc. In our study, samples containing varying concentrations of ^99m^Tc-RP were obtained by spiking with adequate quantities of sodium pertechnetate and measured with a validated method for comparison with the proposed alternative counting method. Seetharaman et al. used a similar approach to determine linearity of their method (Seetharaman et al. 2006).

### Accuracy

Accuracy can be described as being as near as possible to the expected value. For the experiments in this study, we used a similar approach to Mambilima, comparing differences between values from standard to proposed methods (Mambilima 2016). We calculated the difference between RCP mean value of the standard method and the method of study for the three radiopharmaceuticals at each RCP level after spiking with pertechnetate. All differences in RCP were smaller than 1.5%, confirming the method of study met the accuracy criteria. Different approaches have been used in the literature. Mihon et al. used the percentage recovery within a predetermined specified range to demonstrate the accuracy of the method he proposed (Mihon et al. 2016), while Leonardi et al. in 2012 used the RSD value of the mean percentage recovery to validate the accuracy of their method (Leonardi et al. 2012). As we used a TLC method here, the recovery parameter is not relevant, because all used sample material will remain present during the analytical procedure, which is not the case for methods using chromatography columns like HLPC.

In this study HMDP, DMSA and MIBI accuracy analysis met the acceptance criteria at the five RCP levels tested. Thus for the three radiopharmaceuticals the comparison of respective results obtained from the two counting systems is good. This indicates that the proposed counting method using the RadEye B20 counter is demonstrated to be as good as the Veenstra VCS-203 TLC scanner.

### Precision

Precision can be defined as the closeness of measurements to each other. In contrast with accuracy, no relation is investigated between the result and the expected result, or true value. The validation of precision in the current work was twofold: firstly, we evaluated repeatability with five replicates of a spiked sample for each product. The RSD values of the impurities were much lower than acceptance value 15%. It should be noted that for RCP measurements (i.e. the active pharmaceutical ingredient or radiopharmaceutical compound) such a high RSD value is not acceptable. The proposed method using the RadEye B20 counter is regarded as precise. Secondly, we did intermediate precision tests by the evaluation of the method using different operators, and analysis at different time points, and found no significant differences. Seetharaman used a similar approach to compare a standard ITLC method previously developed by Chen et al. (Chen et al. 1993) to a simpler method developed using a solid phase extraction cartridge (Seetharaman et al. 2006). Even though our survey meter is a relatively simple piece of equipment, these results encourage us to advocate the use of the RadEye B20 counter, especially in a low-cost setting.

### Linearity and range

The range of radiochemical purity between approximately 80% and 100% used in the study was chosen in order to include RCP values recommended by manufactures for patient use (95% to 100%) and to be in accordance with ICH and pharmacopeia recommended minimum of five concentrations in the range from 80% to 120% for finished products or assays of drug substances (USP 37 < 1225> 2008). Our samples contained approximately 2 to 3 MBq/5 μl.

To prove linearity, the analytical method should exhibit proportionality to the concentration of the analyte at the chosen range; *r*^2^ should be determined as well as the equation and slope of the regression line. We studied the response of the RadEye B20 counter to Tc-99m radioactivity. Linearity was demonstrated between 0.2 and 5 MBq. As the maximal amount of counts expected for QC strips is much lower than the maximum of the range of the RadEye B20 counter, which can count up to 500 kcps according to its specifications, we expect no dead time problems using our method.

### Robustness

Parameters such as mobile phase composition, and ambient temperature influencing the stationary and mobile phase flow are typically used to evaluate robustness, especially for HPLC methods (Mihon et al. 2016). In this work, we evaluated the suitability of a counting system, which is independent of chemical separation. Careless positioning of the survey meter might lead to differences in counting geometry between the two parts of a chromatography strip. Based on the possibility of slight differences in counting position of the counter, the robustness of the counting system was checked by comparing counts with a correctly positioned counter against a slightly tilted counter. No significant differences in count rates were demonstrated, so the method could withstand at least a small variation in positioning the counter.

### Limit of detection and limit of quantitation

Some authors argue that limits of detection and of quantification are not required for RCP determination with thin layer radiochromatography. Todde et al. indicate that LOQ as usually included in validation studies, may not be relevant where the RCP is determined as a ratio between areas or counts, and no absolute quantitative measurement is required to be performed (Todde et al. 2014). Seetharaman et al. (2006) state that determination of LOD and LOQ can be omitted as only products with RCP values higher than 90% are used clinically. It is however important to know if the counting device is able to distinguish the lowest expected or clinically meaningful impurity activity from background counts.

Tc-99 m products are usually required to have a RCP of 90 or 95%, i.e. not more than 5% of impurities may be present. Exact quantification of impurities larger than 2% may not be mandatory, as it will not affect the acceptance of the product. Calculated from our linearity measurements, 2% of our 5 MBq samples would give a count rate of approximately 80 cps, or 50 times the LOQ of the survey meter. Looking at the calibration curve, uncertainties are present in the lower range of radioactivity. We think that small impurities will rather be overestimated than underestimated. Thus the survey meter can be used, but care should be taken not to use very low amounts of Tc-99 m, as impurities might then be below the LOQ.

### Specificity

The specificity of chromatographic methods depends on the separation of different chemical entities, e.g. the active pharmaceutical ingredient and any impurities. For HPLC, this means that the peaks of the different chemical entities should be clearly separated. In our evaluation of the method of quantification of the distribution of radioactivity, the chemical separation was done with thin layer chromatography, and the same strip was then used for both counting methods. We therefore did not include specificty in our validation. For the same reason we also did not test for impurities other than pertechnetate.

### Summary

Guidelines for analytical method validation as presented by international regulatory bodies and national authorities (e.g. ICH, FDA) and described in available literature, usually do not provide much information for radioanalytical methods such as radio-TLC (ICH Q2R1, EMEA 2006). A notable exception is the Guide published by the EDQM in 2018 which does address validation of analytical methods for radiopharmaceuticals. Using internationally recommended parameters, we validated our proposed method by comparison with a validated method routinely used in a GMP unit.

Limitations of this study include the non-availability of reference standards for Tc-99 m labelled compounds since there are no stable isotopes of technetium (Todde et al. 2014). The range over which the linearity curve for the survey meter was determined, includes typical activities for sample drops used for RCP analysis of Tc-99m radiopharmaceuticals with iTLC, but should be extended at the lower end to unequivocally include activities expected in impurities representing 0.5% of the sample. Furthermore the linearity measurement with the survey meter for Tc-99m should be extended to include higher activities if radiopharmaceuticals with higher radioactivity concentrations are to be analysed.

In validation studies, sample preparation should be done very carefully, and effects like possible adhesion of impurities to containers used in preparing samples should be considered in the design of the tests. Furthermore, spiking samples with pertechnetate may result in additional labeling of the Tc-99m-radiopharmaceutical, which may cause an additional uncertainty in the outcome of the experiments.

## Conclusion

Compared to the standard method, our alternative method was linear, accurate, specific in the range of product concentrations internationally recommended for patients, precise, and robust. The value of the successful validation of the proposed method is twofold: Firstly, it has increased the awareness of the importance of validation among staff in our unit, and secondly, a cost-effective method is now available and can be used in any other low income Nuclear Medicine units.

## Abbreviations

ANOVA: Analysis of variance
cps: Counts per second
CV: Coefficient of variation
DMSA: Dimercaptosuccinic acid
FDA: Food and Drug Administration
GMP: Good manufacturing practice
HMDP: Hydroxymethylene diphosphonate
HPLC: High-Performance Liquid Chromatography
IAEA: International Atomic Energy Agency
ICH: International Council for Harmonisation
ISO: International Organization for Standardization
iTLC-SG: Instant thin layer chromatography- silica gel
keV: kilo electron Volt
LOQ: Limit of quantitation
MIBI: Methoxyisobutylisonitrile
PET: Positron Emission Tomography
QA: Quality assurance
QC: Quality control
RCP: Radiochemical purity
RP: Radiopharmaceutical
RSD: Relative standard deviation
SPECT: Single Photon Emission Computed Tomography
TLC: Thin layer chromatography
UMCG: University Medical Center Groningen
UPLC: Ultra Performance Liquid Chromatography
USP: United States Pharmacopoeia
YGH: Yaoundé General Hospital
